# High Concentrations of Non-Esterified Fatty Acids During Bovine *In Vitro* Fertilisation Are Detrimental for Spermatozoa Quality and Pre-Implantation Embryo Development

**DOI:** 10.3390/jdb13040035

**Published:** 2025-10-05

**Authors:** Abdullah F. Idriss, Edward J. Okello, Roger G. Sturmey, Miguel A. Velazquez

**Affiliations:** 1 Translational and Clinical Research Institute, Newcastle University, Newcastle upon Tyne NE1 7RU, UK; aidriss97@kfshrc.edu.sa (A.F.I.); edward.okello@newcastle.ac.uk (E.J.O.); 2 Biomedical Institute for Multimorbidity, Centre for Biomedicine, Hull York Medical School, University of Hull, Hull HU6 7RX, UK; roger.sturmey@hyms.ac.uk; 3 School of Natural and Environmental Sciences, Newcastle University, Newcastle upon Tyne NE1 7RU, UK

**Keywords:** non-esterified fatty acids, fertilisation, spermatozoa quality, pre-implantation embryo development, H3K27me3

## Abstract

High non-esterified fatty acids (NEFAs) during negative energy balance in dairy cattle can impair reproduction. While their effects on oocyte maturation and preimplantation embryo development are known, their impact during fertilisation is largely unexplored. This study examined the effects of high NEFA exposure exclusively during in vitro fertilisation (IVF). Bovine oocytes were matured in vitro and fertilised under physiological or high NEFA concentrations. High NEFA concentrations decreased fertilisation, cleavage, and blastocyst rates. Reactive oxygen species production in zygotes was not affected, but blastocysts derived from the High-NEFA group had fewer cells. Spermatozoa exposed to high NEFA concentrations exhibited increased plasma membrane and acrosome damage, higher DNA fragmentation, and reduced mitochondrial membrane potential. The expression of H3K27me3, a repressive histone mark normally erased from fertilisation to embryonic genome activation, was higher in 2-cell than in 4-cell embryos on day 2 after IVF, but only in the High-NEFA group. This delayed H3K27me3 loss, along with increased DNA damage, could partially explain the reduced blastocyst formation observed. In conclusion, high NEFA concentrations can impair pre-implantation embryo development during zygote formation, potentially via effects on both the oocyte and spermatozoon. The latter warrants further investigation using an intracytoplasmic sperm injection model.

## 1. Introduction

Significant alterations in hormones and systemic metabolites occur during human obesity and in dairy cattle experiencing a negative energy balance (NEB). These metabolic shifts often disrupt normal physiological processes and can compromise reproductive function. Among the many metabolic changes observed, elevated non-esterified fatty acids (NEFAs) (also known as free fatty acids) have been identified as particularly influential. Increased NEFA levels are strongly linked to metabolic stress and have been shown to impair reproductive performance, thereby contributing to subfertility in both obese humans and dairy cows undergoing NEB [1,2,3,4,5]. In vitro bovine models have shown that exposure to NEFA, such as stearic acid (SA), palmitic acid (PA), and oleic acid (OA), at high concentrations during oocyte maturation and embryo development can disrupt both embryo formation and quality. Accordingly, oocytes exposed to high NEFA concentrations during in vitro maturation (IVM) showed impaired DNA methylation of maternal imprinted genes [6], decreased mitochondrial DNA copy number [7], and a reduced ability to reach the blastocyst stage [7,8]. The resultant blastocysts had fewer cells, an increased level of apoptosis, and up-regulation of genes involved in DNA methylation, glucose transport [8] and fatty acid synthesis [9]. Blastocysts derived from high NEFA-exposed oocytes also showed a decreased mitochondrial DNA copy number [7] and an enhanced amino acid metabolism characterised by altered production and consumption of amino acids along with low consumption of oxygen and glucose [8]. Furthermore, transcriptomic and whole-genome DNA methylation analyses carried out in the blastocyst from oocytes exposed to high NEFA concentrations revealed that NEFA can increase the expression of genes in lipid synthesis pathways [10] and altered methylation patterns in loci associated with cellular development, cell death and survival, amino acid metabolism, and cellular growth and proliferation [11]. More recently, transfer of day 7 blastocysts derived from a high NEFA microenvironment during IVM, resulted in the development of embryos with impaired growth when examined 7 days after embryo transfer (i.e., day 14 embryos). The extra-embryonic tissue of day 14 embryos produced with oocytes exposed to high NEFA concentrations during IVM also secreted less interferon tau [12].

Similarly, in vitro exposure to high NEFA concentrations during the preimplantation period can impair blastocyst formation, and embryos that managed to reach the blastocyst stage showed a decreased expression of genes associated with cell–cell interactions, cell growth, and cell differentiation along with altered methylation patterns in loci associated with apoptosis, antioxidant production, and mitochondria dysfunction pathways [11]. However, less is known about the effects of high NEFA during the fertilisation process per se. NEFA can be incorporated by human and bovine spermatozoa [13,14,15,16] and although fatty acids are essential for spermatozoa function [17,18], exposure of spermatozoa to high NEFA concentrations can be detrimental for male fertility [19]. Indeed, in vitro models have shown that human and bovine spermatozoa motility can be decreased by exposure to high NEFA concentrations [20,21]. A previous in vitro fertilisation (IVF) study reported that penetration of bovine spermatozoa into oocytes and the first cell divisions can be impaired when fertilisation takes place under high concentrations of NEFA, but apparently with no effect on blastocyst production. Given that spermatozoa pre-exposed to high NEFA levels managed to achieve fertilisation and that blastocyst formation was not affected, it was suggested that the detrimental effect of NEFA during fertilisation is via the oocyte and not the sperm [21]. However, it is important to assess other relevant indicators of gamete quality, including DNA damage, particularly since spermatozoa with such damage can still achieve fertilisation [22]. Therefore, more research is needed to clarify the role of high NEFA during fertilisation. The aim of this study was to determine the effects of high NEFA exposure exclusively during IVF in cattle to provide new insights on this topic.

## 2. Materials and Methods

### 2.1. Collection of Cumulus Oocyte Complexes

Bovine ovaries collected at a local abattoir were transferred to the laboratory using a warm thermal bag. All Sigma products were obtained from Merck Life Science UK Limited (Sigma-Aldrich, Gillingham, Dorset, UK). At the laboratory, ovaries were washed several times in a warm phosphate-buffered saline (PBS, Sigma P4417) solution (38.5 °C). Any visible, distinctive, large dominant follicles were burst before oocyte collection. Subsequently, ovaries were slashed using scalpels into a glass Petri dish with warm (38.5 °C) oocyte recovery media composed of serum-free tissue culture medium 199 (TCM-199, Sigma M2520) supplemented with 0.1% (*w*/*v*) bovine serum albumin (BSA, fatty acid-free, Sigma A7030), 0.2 mM sodium pyruvate (Sigma P3662), 4.2 mM sodium bicarbonate (Sigma S57761), 50 µg/mL gentamycin sulphate (Sigma G3632), and 10 mM HEPES (Sigma 5310). All media used in this study were prepared with sterile-filtered water (W3500) and sterilised by filtration. Oocyte recovery medium was transferred to a glass beaker and allowed to settle for 15 min at 38.5 °C. Then, the supernatant was aspirated to leave ~100 mL of oocyte recovery medium, which was subsequently aliquoted into 15 mL conical tubes. The precipitate (i.e., oocytes and cellular debris) from each conical tube was aspirated and transferred into a searching plastic Petri dish containing the same pre-warmed oocyte recovery medium. Cumulus oocyte complexes (COCs) of good quality (grade I) with a dark, homogenous ooplasm and at least five layers of compact cumulus cells were selected for IVM [23].

### 2.2. In Vitro Maturation

Selected COCs were matured in groups of 20–30 in a 4-well plate (Nunc^®^, ThermoFisher Scientific, 179820, Paisley, UK) containing serum-free maturation medium (500 µL/well without oil cover) composed of TCM199 medium supplemented with 0.1% (*w*/*v*) BSA, 0.2 mM sodium pyruvate, 26.2 mM sodium bicarbonate, 50 µg/mL gentamycin sulphate, 20 ng/mL murine epidermal growth factor (mEGF, Sigma E4127), and gonadotrophins (Intervet International B.V., Boxmeer, The Netherlands)—5.0 IU human chorionic gonadotrophin (hCG, Chorulon^®^) and 10.0 IU pregnant mare serum gonadotrophin (PMSG-Intervet^®^). COCs were in vitro matured for 22–24 h in a humidified atmosphere with 5% CO_2_, atmospheric O_2_, and at 38.5 °C.

### 2.3. In Vitro Fertilisation

The IVF medium was Fertilisation TALP (Tyrode’s medium base, Albumin, Lactate and Pyruvate) known as Fert-TALP [24]. The medium consisted of 114 mM sodium chloride (Sigma S5886), 3.2 mM potassium chloride (Sigma P5405), 0.3 mM sodium phosphate monobasic dehydrate (Sigma 71500), 2.0 mM calcium chloride dehydrate (Sigma C7902), 0.5 mM magnesium chloride hexahydrate (Sigma M2393), 25 mM sodium bicarbonate, 0.2 mM sodium pyruvate, 10 mM sodium lactate (Sigma L4263), 0.01 µg/mL phenol red (Sigma P5530), 6.0 mg/mL fatty-acid free BSA, 20 µM D-penicillamine (Sigma P4875), 0.1 IU/mL heparin (Sigma H3149), 10 µM hypotaurine (Sigma H1384), 1 µM epinephrine (Sigma H9892), 2 µM sodium metabisulfite (Sigma S9000), and 50 µg/mL gentamycin sulphate.

Sperm straws from a commercial supplier were used for IVF. Sperm straws from two bulls of proven fertility for IVF was thawed in warm water (37 °C) for 1 min and immediately layered on top of 1 mL 90% BoviPureTM (NidaCon International AB, Mölndal, Sweden). The sample was centrifuged (at 300× *g*) for 10 min, and the supernatant was carefully aspirated to leave approximately 50 µL of solution containing the sperm pellet. The solution was then quickly resuspended with 750 µL Fert-TALP without heparin, hypotaurine, and epinephrine and centrifuged for 3 min (at 400× *g*), followed by removal of the supernatant and resuspension with 750 µL of complete Fert-TALP with heparin, hypotaurine, and epinephrine. A third centrifugation was applied for 3 min (at 400× *g*), and the supernatant was removed to leave approximately 50–75 µL of sperm solution. The sperm concentration was calculated with a haemocytometer. COCs were co-incubated in groups of 20–30 with spermatozoa at a final concentration of 1 × 10^6^ sperm cells/mL in a 4-well plate (500 µL/well, without oil cover) for 19 h in a humidified atmosphere with 5% CO_2_, atmospheric O_2_, and at 38.5 °C.

### 2.4. In Vitro Embryo Culture

After 19 h of co-incubation with spermatozoa, the presumptive zygotes were placed in a 1.5 mL Eppendorf tube containing 500 µL of oocyte recovery media and gently vortexed for 4 min to remove excess sperm and cumulus cells. Presumptive zygotes were then quickly washed three times in 500 µL of oocyte recovery media to remove debris after vortexing, followed by three more washes in serum-free modified synthetic oviductal fluid (mSOF), which was the medium used for embryo culture [25]. The mSOF medium contained 4 mg/mL fatty acid-free BSA, 108 mM sodium chloride, 7.2 mM potassium chloride, 1.2 mM potassium phosphate monobasic (Sigma P5655), 1.5 mM magnesium sulphate heptahydrate (Sigma M2643), 5.35 mM sodium lactate, 25 mM sodium bicarbonate, 10 µg/mL phenol red, 7.27 mM sodium pyruvate, 1.78 mM calcium chloride dihydrate, 2.77 mM myo-inositol (Sigma 1.04507), 3.0% (*v*/*v*) basal medium eagle (BME) amino acid solution (50×) (Sigma B6766), 1.0% (*v*/*v*) minimum essential medium (MEM) amino acid solution (100×) (Sigma M7145), 0.2 mM glutamine (Sigma G6392), and 50 µg/mL gentamycin sulphate. After washing, the presumptive zygotes were cultured for 7 days in groups of 20–30 in a 4-well plate (500 µL/well without oil cover) under a humidified atmosphere containing 5% CO_2_ and 5% O_2_, balanced with N2, and at 38.5 °C. On day 8 (day of IVF = day 0), the developmental stage of embryos was classified according to the guidelines of the International Embryo Transfer Society [26] (IETS). The cleavage rate was calculated based on the number of presumptive zygotes cultured and included any embryo with two or more cells on day 8. Degenerated embryos were calculated on the number of cleaved embryos and included any embryo with two or more cells but not able to reach the compacted morula stage on day 8. A cycle of preimplantation embryo production including IVM, IVF, and in vitro embryo culture was considered a replicate.

### 2.5. Preparation of NEFA Treatments

Stearic acid (SA, Sigma S4751) and palmitic acid (PA, Sigma P0500) were dissolved in pre-warmed ethanol (Sigma 51976); while oleic acid (OA, Sigma O1257) was dissolved in pre-warmed cell culture grade water (Sigma W3500). NEFA stocks were prepared at concentrations of 28 mM SA, 23 mM PA, and 21 mM OA for physiological levels and 280 mM SA, 230 mM PA, and 210 mM OA for pathophysiological levels. The stock solutions were vortexed for 15 min and sonicated for 1 h at 35 °C and then added to the serum-free fertilisation medium containing FA-free BSA as the NEFA carrier (to improve NEFA solubility) to obtain the desired final concentration. The fertilisation medium was then sonicated at 35 °C for 4 h and sterilised by filtration prior to IVF.

**Figure 1:**
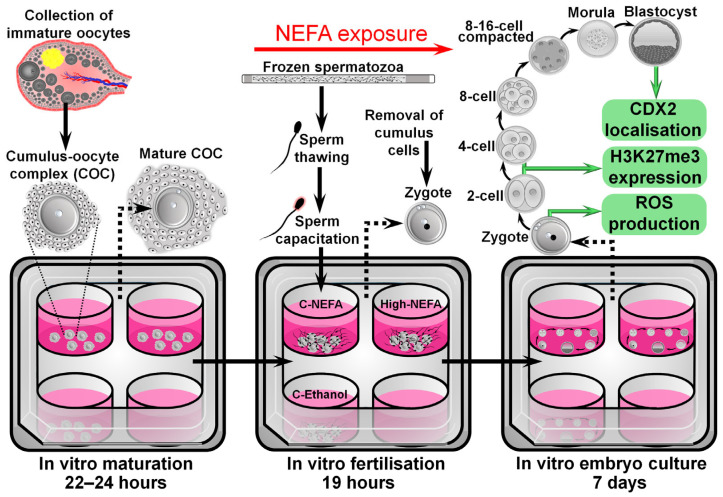
Schematic representation of the experimental design used to analyse the impact of NEFA exclusively during the fertilisation process on pre-implantation embryo development and quality.

The exposure to NEFA concentrations during IVF was designed to approximate what might be expected to be present in the oviduct, the site of fertilisation. The concentrations were based on the model developed by Desmet et al. [21], where NEFA values observed in the serum of cows were considered to be present at the level of the oviduct, since previous research by the same group found a positive correlation between NEFA values in serum and oviductal fluid [27]. As such, a high NEFA group (High-NEFA) containing 280 µM SA, 230 µM PA, and 210 µM OA (720 µM total NEFA), representing pathophysiological NEFA concentrations present in cows undergoing negative energy balance, was compared to two control groups. One control group (C-NEFA) contained 28 µM SA, 23 µM PA, and 21 µM (72 µM total NEFA), representing physiological NEFA concentrations observed in cows out of the phase of negative energy balance. The second control group contained fertilisation medium with 0.2% absolute ethanol, equivalent to the same concentrations as the NEFA groups (C-Ethanol) (Figure 1).

### 2.6. Analysis of Reactive Oxygen Species (ROS) in Zygotes

Presumptive zygotes were cultured in groups of 20–30 in a 4-well plate containing mSOF medium (500 µL/well) in the presence of CellROX™ Green (ThermoFisher Scientific, C10444, Paisley, UK) at a concentration of 5 µM [28] and Hoechst 33342 (5 µg/mL, Sigma B2261) for 30 min in a humidified atmosphere, under 5% CO_2_ and 5% O_2_, balanced with N_2_, and at 38.5 °C. Afterwards, zygotes were washed in 0.1% *w*/*v* polyvinylpyrrolidone (PVP, Sigma P0930) in PBS to remove mSOF culture media and subsequently fixed with 4% formaldehyde in PBS for 15 min at room temperature (18–20 °C). Zygotes were then washed in 0.1% *w*/*v* PVP in PBS to remove fixation medium. Finally, presumptive zygotes were mounted onto a glass microscope slide with double enforcement rings in a small drop of glycerol medium (~5 µL), covered with a cover slip, sealed with nail varnish, and kept in a fridge until analysis of fluorescence intensity. The florescence intensity of CellROX and number of nuclei were analysed on the day of staining. Digital photographs of presumptive zygotes were obtained in a darkened room with an epifluorescence microscope (Zeiss Axio Imager, Carl Zeiss Optics Co., Ltd., Oberkochen, Germany) equipped with a digital camera (Zeiss Axiocam 105) using a 40× objective lens with DAPI filter (excitation 360–370 nm, emission 420–460 nm; for Hoechst fluorescence) and Alexa 488 filter (excitation 460–490, emission 500–520 nm for CellROX™ Green Fluorescence). To quantify ROS levels in a standard area in all zygotes (to control for oocyte size), a circular area of 2500 µm^2^ was drawn with Zeiss software (Zen Blue Imaging, 3.1) in the middle of the zygote, and an identical area was also drawn on the black background. The fluorescence intensity was then measured by subtracting the black background intensity from the green fluorescence intensity. The number of pronuclei was also quantified to determine the fertilisation status. Overlapping or out of focus pronuclei in the digital photographs were identified by manual focus scanning of the samples under the microscope at the time of imaging. The same microscopy settings were used for all presumptive zygotes.

### 2.7. Analysis of Cell Allocation in Blastocysts

Day 8 zona-intact blastocysts were washed with 0.1% PVP/PBS and subsequently fixed in 4% *v*/*v* formaldehyde (Sigma F8775) in PBS for 15 min at room temperature. The fixing solution was then removed by washing embryos in 0.1% *v*/*v* tween (Sigma P9416) in PBS (tween/PBS). After washing, blastocysts were permeabilised and blocked in a solution containing 3% *w*/*v* BSA and 0.5% *v*/*v* triton (Sigma 648464) in PBS for 1 h at room temperature. After blocking and permeabilisation, blastocysts were washed in tween/PBS followed by incubation with a ready-to-use anti-CDX2 primary antibody (Abcam, ab227201, Cambridge, UK) at a 1:200 dilution in tween/PBS overnight at 4 °C. The next day, blastocysts were washed in tween/PBS and incubated with goat anti-rabbit secondary antibody (ThermoFisher Scientific, R37116, Paisley, UK) at a 1:500 dilution in tween/PBS for 30 min at room temperature. The antibody solution was then removed by washing in tween/PBS, and blastocysts were incubated with DAPI (10 ng/mL, Sigma D9542) in tween/PBS for 30 min at room temperature. After a final wash, blastocysts were mounted on a microscope slide within double reinforcement rings in a small drop of Citifluor (Electron Microscopy Sciences, AF1 17970, Hatfield, PA, USA) anti-fading medium (~5 µL), covered with a cover slip, sealed with nail varnish, and stored at 4 °C until analysis. Embryos were analysed on the same day they were mounted on microscope slides. Negative controls were treated as described above except that exposure to the anti-CDX2 primary antibody was omitted. Embryos were examined in a dark room with a confocal laser scanning microscope equipped with a Zeiss LSM 800 Airyscan microscope (Carl Zeiss Optics Co, Ltd., Oberkochen, Germany) running the Zen Blue Imaging 3.1 software and a Plan-Neofluar 25/0.8 objective lens. A solid-state laser was used to detect DAPI (405 nm excitation) and Alexa AF488 (488 nm excitation) using GaAsP detectors and a 400–650 nm bandpass, switching sequentially between channels. Optical sections were taken at 1 µm intervals across the whole embryo using a 37 µm pinhole (equivalent to a 5.8 µm optical section). The cell number in blastocysts was obtained manually using IMARIS software v9.8.2 (Oxford Instruments, Abingdon, UK). The software allows three-dimensional (3D) visualisation of the entire preimplantation embryo and provides specific channel visualisation to quantify CDX2-positive cells, representing cells in the trophectoderm and DAPI-stained cells. The software also allows 3D rotation of images, which facilitates the counting of cells at different angles of the embryo, providing a thorough examination of each blastocyst. The number of cells in the inner cell mass was calculated by subtracting the CDX2-positive cell number from the total cell number (i.e., DAPI-stained cells) in each embryo.

### 2.8. Immunofluorescence Assay for Tri-Methylation of Histone H3 at Lysine 27 (H3K27me3)

Two- and four-cell embryos were collected on day 2 (i.e., 48 h post-fertilisation) and immediately washed in 0.1% PVP/PBS and then fixed with 4% formaldehyde/PBS for 15 min at room temperature. Embryos were then washed in 0.1% tween/PBS, followed by permeabilisation in 1% triton/PBS for 30 min at room temperature. Permeabilisation solution was removed by washing embryos in tween/PBS and embryos were then blocked for two hours at room temperature in 10% goat serum (Sigma G9023), followed by washing in tween/PBS. Then, embryos were incubated with anti-H3K27me3 primary antibody (Abcam, ab6002, Cambridge, UK) [29] at a 1:50 dilution in tween/PBS, in the dark, and the 4-well plate containing the embryos was placed on a microplate shaker overnight at 4.0 °C. The next morning embryos were washed five times (10 min each at room temperature) with tween/PBS and then incubated with goat anti-mouse secondary antibody (Abcam, ab205719, Cambridge, UK) at a dilution of 1:100 in tween/PBS, in the dark. The 4-well plate containing the embryos was placed on a microplate shaker for 30 min at room temperature. After washing with tween/PBS, embryos were incubated for 30 min at room temperature with DAPI. Embryos were then washed and mounted on a glass slide with double reinforcement rings in a small drop of Citifluor anti-fading medium, covered with a coverslip, sealed with nail varnish, and stored at 4 °C in the dark until analysis. Embryos were analysed on the same day they were mounted on microscope slides. Negative controls were treated as described above except that exposure to the anti-H3K27me3 primary antibody was omitted. Embryos were examined with confocal microscopy as described for CDX2, with the exception that optical sections were taken at 7.59 µm intervals across the whole embryo using a 100 µm pinhole (equivalent to a 15.2 µm optical section). The fluorescence intensity for H3K27me3 was measured in IMARIS software. In 3D mode, the diameter of individual nuclei in the blue channel (DAPI) was indicated to delineate nuclei on each embryo. Then, in surface tool mode, the fluorescence intensity in arbitrary units was provided by the software for each delineated nucleus. The fluorescence in the green channel was used to determine the levels of H3K27me3. The mean fluorescence value from the total number of blastomeres on each embryo was used for statistical analysis.

### 2.9. Evaluation of Spermatozoa Quality

**Figure 2:**
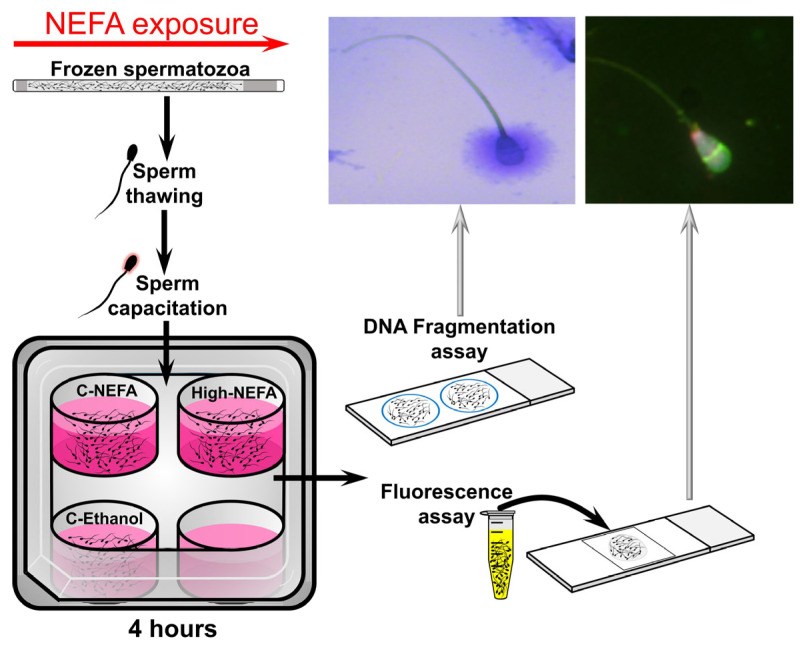
Schematic representation of the experimental design used to analyse the impact of NEFA on spermatozoa quality.

Straws of cryopreserved spermatozoa were thawed and processed to obtain a concentration of 25 × 10^6^ sperm/mL (for fluorescent assay) or 20 × 10^6^ sperm/mL (for DNA fragmentation assays) in Fert-TALP medium. A volume of 500 µL in a 4-well plate (500 µL/well, without oil cover) was used to expose spermatozoa to the same NEFA concentrations used under IVF conditions (i.e., at 38.5 °C in a humidified atmosphere with 5% CO_2_, and atmospheric O_2_). Spermatozoa were exposed to NEFA for 4 h, followed by analyses of acrosome and plasma membrane integrity, mitochondrial membrane potential, and DNA fragmentation. A cycle of thawing, NEFA exposure, and spermatozoa analyses was considered a replicate (Figure 2). 

Fluorescence assays were used to simultaneously evaluate acrosome integrity, mitochondrial membrane potential, and plasma membrane integrity in spermatozoa. The protocol used was based on the technique developed by Celeghini et al. [30] with some modifications. Staining probes used to examine spermatozoa were as follows:

Hoechst H33342 was used to identify spermatozoa with an undamaged plasma membrane as the dye is cell permeant and is often used to stain DNA in living cells (or fixed cells) [31]. A stock solution of 25 mg/mL (50 mM) in dimethyl sulphoxide (DMSO) (Sigma D8418) was used to prepare a working solution of 40 µg/mL in Dulbecco’s phosphate buffered saline (DPBS) (Sigma D5652) and stored in 10 µL aliquots at −20 °C.

DRAQ7™ (Biostatus DR70250, Shepshed, UK) was used to identify spermatozoa with a damaged plasma membrane, as the dye does not enter cells with intact plasma membrane integrity [32]. A working solution of 0.3 µM in DPBS was prepared and stored in 10 µL aliquots at 4 °C [33].

Lectin from Pisum sativum (Pisum sativum agglutinin [PSA]) was labelled with fluorescein isothiocyanate (FITC) (Sigma L0770, PSA, FITC conjugate). FITC-PSA was used to determine the acrosomal status, as PSA binds acrosome-reacted spermatozoa. As such, a spermatozoon showing the acrosomal region completely or partly marked with FITC-PSA was considered to be acrosome-reacted [34] and an indication of acrosome damage [30]. A working solution was prepared containing 1 mg/mL FITC-PSA in DPBS and stored in 75 µL aliquots at 4 °C.

For 5,5′,6,6′-tetrachloro-1,1′,3,3′-tetraethylbenzimidazolyl carbocyanine iodide (JC-1) (ThermoFisher Scientific, T3168, Paisley, UK), the JC-1 probe allows detection of changes in mitochondria membrane potential (Δψm) by changes in the colour of the dye, turning from green (i.e., low Δψm) to red-orange (high Δψm) as polarisation of the mitochondrial membrane increases [35,36]. A stock solution of 1.53 mM JC-1 in DMSO was used to prepare a working solution of 153 µM JC-1 in DMSO that was stored in 10 µL aliquots at −20 °C.

All staining probes and plasticware were pre-warmed prior to use (at 37 °C). Following NEFA exposure in 500 µL of Fert-TALP medium, 150 µL of sperm suspension were aspirated and transferred into a 0.5 mL Eppendorf tube, where 2 µL of H33342 were added, and the resultant sperm suspension was incubated for 10 min at 37 °C. The sperm suspension was then centrifuged (100× *g* for 1 minute), with the supernatant then removed, leaving 60–70 µL of sperm solution. Then, 100 µL of Fert-TALP medium was added, plus 3 µL of DRAQ7, 2 µL of JC-1, and 50 µL of FITC-PSA, followed by incubation at 37 °C for at least 8 min. The sperm suspension was then centrifuged (100× *g* for 1 minute), and the supernatant was removed to leave 60–70 µL of sperm suspension, to which 40 µL of Fert-TALP medium was added. From this final sperm suspension, an 8 µL drop was placed on a microscope slide, covered with a cover slip, and evaluated immediately with an epifluorescence microscope equipped with a digital camera under a 63× oil objective lens with a multi-bandpass filter for visualisation of Hoechst 33342 (excitation 352 nm and emission 454 nm), DRAQ7 (excitation 598 nm and emission 697 nm), FITC-PSA (excitation 498 nm and emission 518 nm), and JC-1 (excitation/emission 514/529 nm for the monomer and 585/590 nm for the J-aggregate). Four biological replicates were applied for each treatment group, and two hundred sperm cells per replicate were examined and classified manually using the Zeiss ZEN microscopy software.

Sperm DNA damage was assessed with the sperm chromatin dispersion assay [37]. A commercial kit (Halosperm G2^®^ kit, Halotech DNA, S.L., Madrid, Spain) previously used in bovine spermatozoa [38] was applied to NEFA-treated spermatozoa according to the manufacturer’s instructions. The agarose screw tube provided in the kit was placed in a water bath at 95–100 °C for 5 min to melt the agarose, which was then divided into 100 µL aliquots in 0.5 Eppendorf tubes. One tube was used per group. and the rest of the tubes were kept at 4 °C for future use. The tubes to be used were kept at 37 °C for 5 min, and then 50 µL of the 500 µL sperm suspension used for NEFA exposure was added to the tube and mixed gently with a pipette, making sure no bubbles were formed. Then, 8 µL of the sperm/agarose suspension was immediately placed in the well of the coated microscope slides provided in the kit (two wells per slide) and covered with a cover slip. The slides were placed on a pre-cooled glass plate and moved to a refrigerator to keep the slides at 4 °C for 5 min to allow solidification of the agarose containing the spermatozoa. Then, out of the refrigerator, cover slips were removed by sliding them gently off the slides. For the rest of the protocol, the procedure was done at room temperature (18–20 °C), with slides kept horizontal at all times, and solutions, water, and ethanol removed by gently tilting the slides, without shaking them. Slides were placed in an elevated position inside a Petri dish and solution 1 (i.e., the denaturant agent) of the kit was added and left to incubate for 7 min, making sure it covered the wells in the slides entirely. Solution 1 was then removed and solution 2 (i.e., lysis solution) was added, making sure the wells were immersed in the solution. After 20 min of incubation, solution 2 was removed and the slides were washed with distilled water for 5 min. Water was then removed, and samples were dehydrated with a two-step exposure to ethanol (2 min each), first with 70% and then with 100%. Following removal of ethanol and air drying of samples, a 7- to 10-min incubation with solution 3 (i.e., eosin staining solution) was done, making sure the solution covered the wells completely. Solution 3 was removed, and solution 4 (i.e., thiazine staining solution) was applied in the same way as solution 3. After removal of solution 4, slides were air-dried, and wells were covered with cover slips and analysed with bright-field microscopy using a 40× objective. Spermatozoa with a big or medium-sized halo were considered normal, without fragmented DNA, whereas spermatozoa with a small or no halo and exhibiting degeneration were considered to have fragmented DNA. Approximately 150 spermatozoa were counted per slide-well. Six biological replicates were performed to examine DNA fragmentation. For the positive control, (i.e., all spermatozoa display a halo), all protocol steps were applied except adding the denaturant agent (solution 1), while for the negative control. (i.e., all spermatozoa do not show a halo), only the lysis reagent (solution 2) was omitted.

### 2.10. Statistical Analysis

Statistical analysis was performed with SPSS 26 software (IBM). The Shapiro–Wilk test was used to test the normal distribution of data. Differences between groups were identified using analysis of variance (ANOVA) or *t*-test, with percentage data arcsine transformed before analysis. A *p*-value less than 0.05 was considered statistically significant. Post-hoc comparisons (for ANOVA) were done with the least significant difference (LSD) method. Data were reported as the mean ± standard error of the mean (SEM) unless otherwise indicated.

## 3. Results

### 3.1. Exposure to High Concentrations of NEFA Exclusively During Fertilisation Decreases Sperm Penetration into the Oocyte but It Does Not Impact ROS Levels in the Resultant Bovine Zygotes

**Figure 3:**
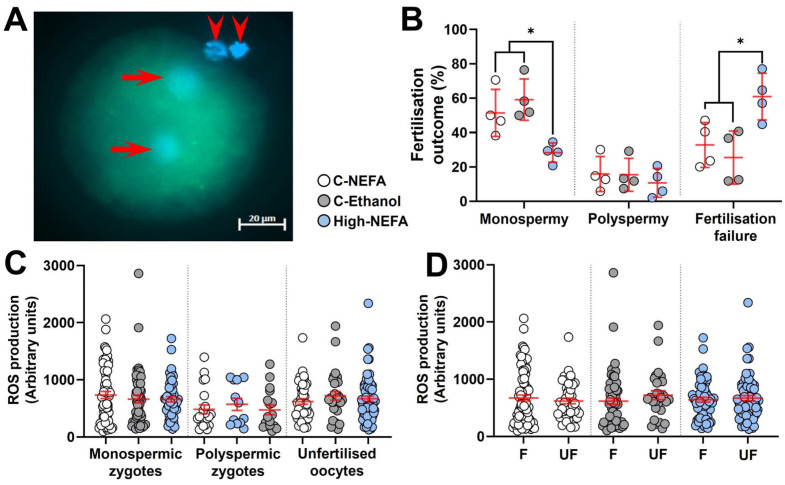
Impact of high concentrations of NEFA during IVF on fertilisation outcomes and reactive oxygen species (ROS) generation in resultant bovine zygotes. (**A**) Representative ROS fluorescence in a monospermic zygote. Arrows indicate pronuclei, and arrow heads show polar bodies. (**B**) Effect of NEFA exposure on fertilisation outcomes. Presumptive zygotes (High-NEFA *n* = 129, C-NEFA *n* = 128, C-Ethanol *n* = 98) from 4 biological replicates were analysed. Each data point corresponds to the average value from a single biological replicate. (**C**) ROS production in relation to fertilisation status and treatment. (**D**) ROS production in fertilised (F, i.e., monospermic plus polyspermic zygotes) and unfertilised (UF) oocytes in each treatment group. In figures **C** and **D** each data point corresponds to an individual zygotic ROS value. An asterisk (*) indicates a significant difference between groups (*p* < 0.05).

Analysis of the pronuclear number in presumptive zygotes (Figure 3A) revealed that the percentage of monospermic fertilisation was significantly decreased in the High-NEFA group (28.32 ± 2.82%) compared to both C-NEFA (51.41 ± 6.86%, *p* = 0.026) and C-Ethanol (59.16 ± 6.04%, *p* = 0.006). However, there was no significant difference in the polyspermy rate among the treatment groups (C-NEFA: 15.84 ± 5.09%, C-Ethanol: 15.42 ± 4.75%, High-NEFA: 10.74 ± 4.18%). The percentage of unfertilised oocytes was higher in the High-NEFA group (60.94 ± 6.76%) than in both control groups (C-NEFA: 32.75 ± 6.53%, *p* = 0.017; C-Ethanol: 25.42 ± 7.72%, *p* = 0.006) (Figure 3B). The ROS fluorescence intensity was not affected by high NEFA concentrations in monospermic or polyspermic zygotes or in unfertilised oocytes (Figure 3C). Similarly, within groups, ROS production levels did not differ between fertilised (monospermic and polyspermic zygotes) and unfertilised oocytes, indicating that fertilisation status per se did not affect ROS production (Figure 3D). There were no significant differences between the control groups in any of the fertilisation outcome variables analysed or in ROS measurements in zygotes.

### 3.2. Exposure to High Concentrations of NEFA Exclusively During Fertilisation Impairs Bovine Pre-Implantation Embryo Development and Cell Allocation of Resultant Blastocysts

**Figure 4:**
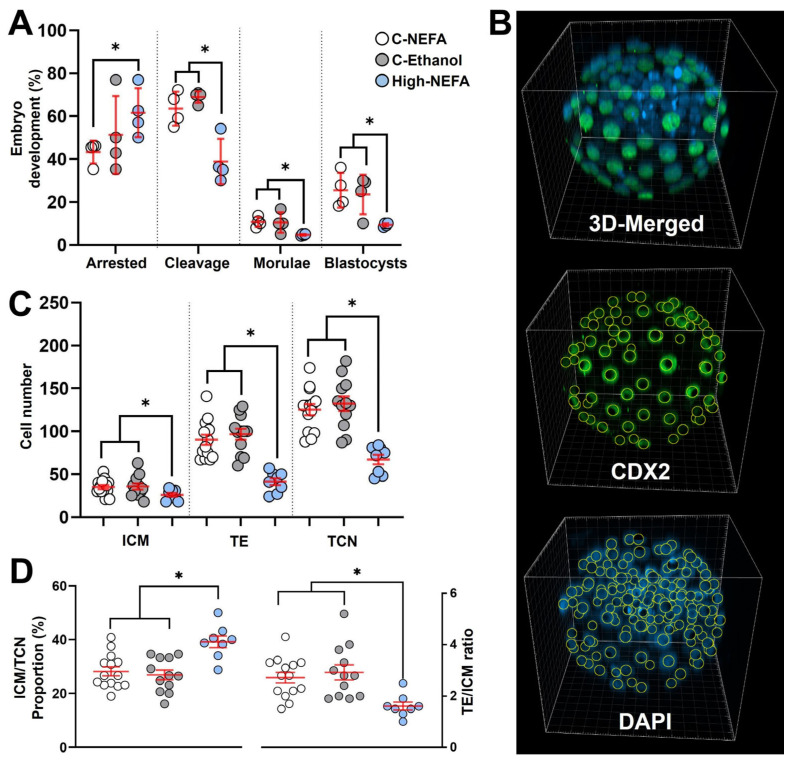
Impact of high concentrations of NEFA during IVF on pre-implantation embryo production and cell lineage allocation in resultant bovine blastocysts. (**A**) Effect of NEFA exposure on in vitro embryo development. Presumptive zygotes (High-NEFA *n* = 86, C-NEFA *n* = 85, C-Ethanol *n* = 84) from 4 biological replicates were analysed. Each data point corresponds to the average value from a single biological replicate. (**B**) Representative IMARIS software display illustrating the setup used to examine cell lineage allocation in blastocysts via 3D screening with confocal microscopy. CDX2 staining specifically marks the trophectoderm (green), while DAPI stains all nuclei within the blastocyst (blue). (**C**) Cell number in blastocysts derived from high NEFA exposure during zygote formation (High-NEFA *n* = 8, C-NEFA *n* = 14, C-Ethanol *n* = 12). Total cell number (TCN) and the cell lineages comprising the trophectoderm (TE) and inner cell mass (ICM, DAPI-stained cells not expressing CDX2) were quantified. (**D**) Blastocyst lineage specification metrics. In figures (**C**,**D**) each data point corresponds to the value of an individual embryo. An asterisk (*) indicates a significant difference between groups (*p* < 0.05).

Exposure to high concentrations of NEFA decreased the cleavage rate (46.16 ± 2.95%) compared to both controls (C-NEFA: 64.97 ± 5.06%, C-Ethanol: 68.96 ± 1.33%, *p* = 0.006). Embryo arrest occurred at a higher rate in the High-NEFA group (66.52 ± 3.73%) than in the C-NEFA group (42.44 ± 2.49%, *p* = 0.046), although the difference with C-Ethanol (49.35 ± 7.28%) did not reach statistical significance (*p* = 0.052). Morula formation was higher in the control groups (C-NEFA: 10.69 ± 1.18%, *p* = 0.033; C-Ethanol: 10.42 ± 2.39%, *p* = 0.014) relative to the High-NEFA group (4.68 ± 0.20%). Similarly, blastocyst formation was more prevalent in the control groups (C-NEFA: 25.49 ± 4.08%, C-Ethanol: 23.54 ± 4.64%) than in the High-NEFA group (9.36 ± 0.40%, *p* < 0.001). No significant differences were observed between the control groups for any of the embryo production variables analysed (Figure 4A).

Three-dimensional quantitative imaging of generated blastocysts (Figure 4B) revealed that embryos derived from high NEFA exposure had significantly fewer cells in the trophectoderm (TE) (41.3 ± 4.1) compared to those from C-NEFA (90.2 ± 5.9, *p* = 0.001) and C-Ethanol (96.7 ± 6.4, *p* < 0.001) groups. Similarly, the cell number in the inner cell mass (ICM) of blastocysts from the High-NEFA group (26.0 ± 2.2) was lower than in the control groups (C-NEFA: 35.0 ± 2.4, C-Ethanol: 36.0 ± 3.5, *p* < 0.001). This resulted in a decreased total cell number (TCN) in high NEFA-derived blastocysts (67.3 ± 5.6) compared to embryos in control groups (C-NEFA: 125.2 ± 6.6, C-Ethanol: 132.3 ± 8.4, *p* < 0.001) (Figure 4C). Examination of the ratio of TE to ICM showed a decrease in the High-NEFA group (1.6 ± 0.2) compared to C-NEFA (2.7 ± 0.2, *p* < 0.001) and C-Ethanol (2.9 ± 0.3, *p* < 0.001). 

Further analysis of cell allocation also revealed that the proportion of cells allocated to the ICM (ICM/TCN) was increased in high NEFA-derived blastocysts (39.1 ± 2.2%) in comparison with controls (C-NEFA: 28.2 ± 1.7%, C-Ethanol: 26.9 ± 1.8%, *p* < 0.001) (Figure 4D). Blastocysts with an ICM/TCN proportion higher than 40% were present only in the High-NEFA group, comprising 25% (2 of 8) of the blastocysts examined. Cell allocation variables did not differ significantly between the control groups (Figure 4C,D).

### 3.3. Exposure to High Concentrations of NEFA Exclusively During Fertilisation Delays the Programmed Loss of Histone Mark H3K27me3 Between the First and Second Cleavage Stages

**Figure 5:**
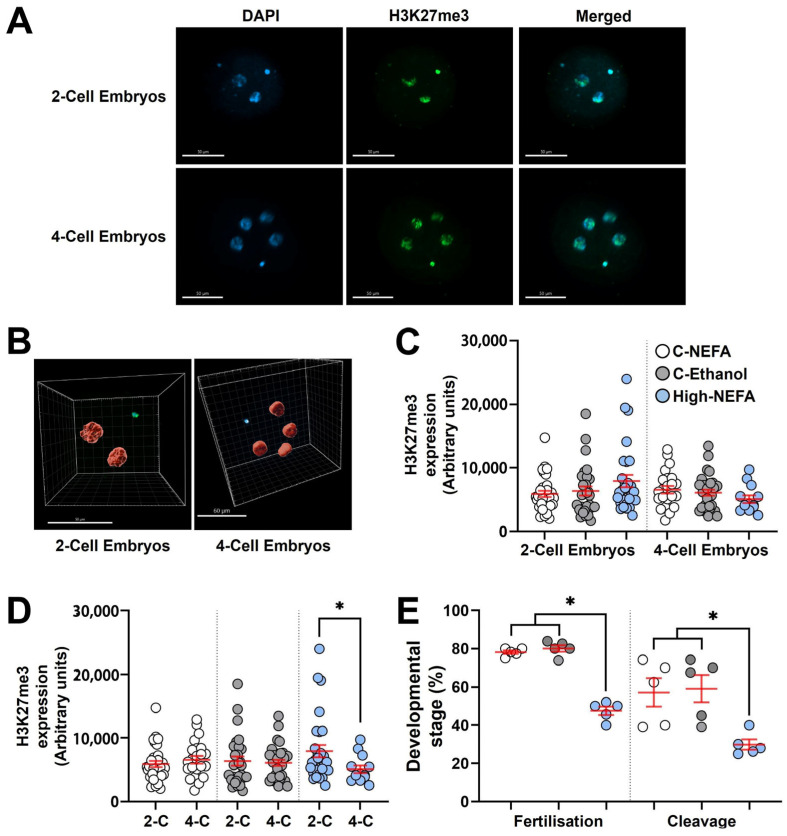
Impact of high concentrations of NEFA during IVF on the expression of H3K27me3 in early bovine embryos. Two-cell (High-NEFA *n* = 29, C-NEFA *n* = 31, C-Ethanol *n* = 29) and four-cell (High-NEFA *n* = 13, C-NEFA *n* = 32, C-Ethanol *n* = 23) embryos were analysed in five biological replicates. (**A**) Confocal microscopy images of equatorial optical sections of embryos showing nuclear localisation of H3K27me3 protein in 2-cell and 4-cell embryos. (**B**) Representative IMARIS software display illustrating the setup used to examine H3K27me3 protein expression via 3D measurement with confocal microscopy. (**C**) Expression of H3K27me3 in 2-cell and 4-cell embryos in relation to experimental treatments. (**D**) Comparison of H3K27me3 expression between 2-cell (2-C) and 4-cell (4-C) embryos in each experimental group. In figures **C** and **D** each data point corresponds to the value of an individual embryo. (**E**) Fertilisation and cleavage rates in biological replicates used for analysis of H3K27me3 expression. Each data point corresponds to the average value from a single biological replicate. An asterisk (*) indicates a significant difference between groups (*p* < 0.05). Scale bars are 50 µm for figure **A** and for 2-cell embryos in figure **B**, and 60 µm for 4-cell embryos in figure (**B**).

Analysis of H3K27me3 expression (Log10 transformed data) between groups using 3D imaging (Figure 5A,B) indicated that exposure to elevated NEFA concentrations during zygote formation did not significantly alter H3K27me3 abundance in embryos at either the 2-cell or 4-cell stage (Figure 5C).

Both 2-cell and 4-cell embryos were collected 48 h post-fertilisation (hpf). Since the decrease in H3K27me3 is not due to dilution of epigenetic marks during cleavage, but instead reflects a cell division–independent process [29], and given that embryos remaining at the 2-cell stage around 40–46 hpf are expected to exhibit H3K27me3 levels similar to 4-cell embryos collected at the same time point [29,39], a subsequent analysis was performed to compare H3K27me3 levels between 2-cell and 4-cell embryos within each experimental group. This intra-group analysis revealed that 2-cell embryos exhibited significantly higher H3K27me3 expression than 4-cell embryos exclusively in the High-NEFA group (*p* = 0.038). This difference in H3K27m3 levels between developmental stages was absent in control groups (Figure 5D).

Fertilisation rate (calculated on cleaved embryos and non-cleaved monospermic zygotes) in the biological replicates used to analyse H3K27me3 expression was significantly decreased under high NEFA conditions (47.54 ± 2.15%) compared to control groups (C-NEFA: 78.14 ± 0.93%, C-Ethanol: 80.06 ± 1.71%, *p* < 0.001). Similarly, the capacity of the resultant zygotes to achieve cleavage was lower in the High-NEFA group (29.8 ± 2.6%) than in the control groups (C-NEFA: 57.1 ± 7.4%, C-Ethanol: 59.1 ± 7.1%, *p* = 0.009). The control groups did not differ significantly in either fertilisation or cleavage rates (Figure 5E).

### 3.4. Exposure to High Concentrations of NEFA Impairs Spermatozoa Quality

**Figure 6:**
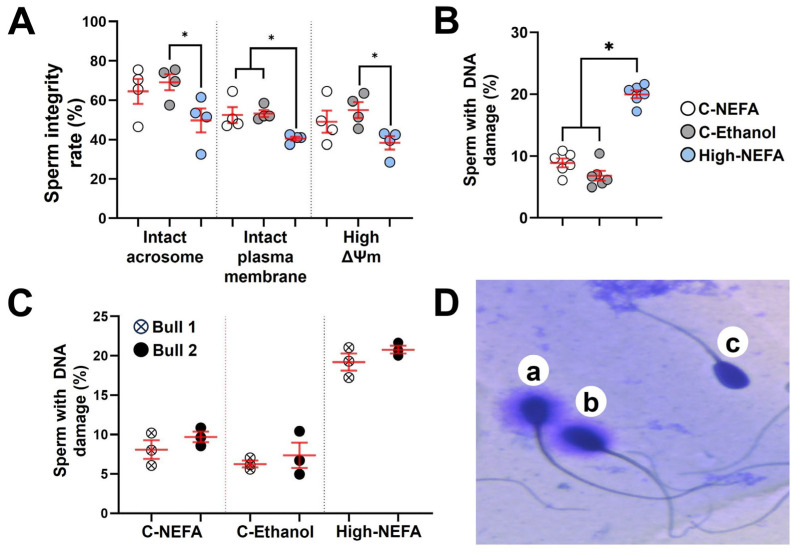
Impact of high concentrations of NEFA on spermatozoa quality. Fluorescence assays were used to simultaneously analyse acrosome and plasma membrane integrity, and mitochondrial membrane potential (ΔΨm) in four replicates (800 sperm cells per group). (**A**) Effect of NEFA on sperm integrity markers. (**B**) Sperm DNA fragmentation following NEFA exposure was analysed in six biological replicates (High-NEFA *n* = 930, C-NEFA *n* = 812, C-Ethanol *n* = 879 sperm cells). (**C**) Comparison of sperm DNA fragmentation in the two bulls used in the assessment of sperm quality following high NEFA exposure across three biological replicates. (**D**) Representative image (light microscopy) of the classification of sperm in the DNA fragmentation assay. Sperm with large (**a**) or medium (**b**) halos were classified as having non-fragmented DNA, while those with small or no halos (**c**) were considered to have fragmented DNA. For all graphs, each data point corresponds to the average value from a single biological replicate. An asterisk (*) indicates a significant difference between groups (*p* < 0.05).

The fluorescence assay analysis revealed that spermatozoa in the high-NEFA group exhibited a lower proportion of intact acrosomes (49.75 ± 6.14%) compared to both control groups (C-NEFA: 64.50 ± 6.34%, C-Ethanol: 69.13 ± 4.08%). Despite this trend, the reduction was statistically significant only in comparison with C-Ethanol (*p* = 0.037), while the difference between High-NEFA and C-NEFA did not reach significance (*p* = 0.096). Similarly, a significant decrease in the percentage of spermatozoa with high mitochondrial membrane potential was observed in the High-NEFA group (38.37 ± 3.38%) compared to C-Ethanol (55.00 ± 4.03%, *p* = 0.027), whereas the difference between the High-NEFA group and C-Ethanol (49.12 ± 5.69%) was not statistically significant (*p* = 0.124). The high-NEFA group showed a higher plasma membrane damage (59.50 ± 1.06%) compared to controls (C-NEFA: 47.50 ± 4.07%, *p* = 0.01; C-Ethanol: 46.75 ± 1.79%, *p* = 0.008). The control groups did not have any significant differences in any of the variables analysed in the fluorescence assay (Figure 6A).

Analysis of spermatozoa DNA fragmentation (Figure 6D) showed that high NEFA exposure was associated with elevated levels of DNA damage (20.00 ± 0.64%) compared to control groups (C-NEFA: 8.90 ± 0.71%, C-Ethanol: 6.82 ± 0.78%, *p* < 0.001) (Figure 6B). As previous research suggested that bulls may vary in their susceptibility to sperm DNA damage [40,41], the two bulls used in this study were assessed, and no significant difference was found (Figure 6C). Although the percentage of DNA fragmentation was higher in the physiological NEFA group compared to the ethanol group (Figure 6B), the difference was not statistically significant (*p* = 0.054).

## 4. Discussion

There is extensive evidence indicating that the capacity of an oocyte to promote development to the blastocyst stage is impaired when the oocyte undergoes maturation in a microenvironment with a high NEFA concentration [3,42,43,44]. In contrast, less is known about the effects of high NEFA exposure during the fertilisation process. Our results confirmed the reported decrease in monospermic fertilisation but contrast with the increased level of polyspermy found by Desmet et al. [21]. Interestingly, Desmet et al. [21], using aceto-orcein staining, reported that polyspermy was only observed in oocytes exposed to NEFA, irrespective of the concentration. The absence of polyspermy (0%) in their control group containing solvent appears to be an exception rather than the norm, as polyspermy is a common abnormality during in vitro embryo production in mammals, with reported incidences ranging from 3% to 30% in humans and 5% to 69% in cattle [45,46,47,48]. In cattle, the bull plays a significant role in the risk of polyspermy in vitro, and both high- and low-polyspermy bulls have been reported [47,48,49]. Polyspermy levels in control groups of the present study are within the values reported in low-polyspermy bulls [47,48].

Although fertilisation success was impaired, exposure to high NEFA concentrations during the fertilisation process did not alter ROS production in the resultant monospermic zygotes. It has been reported that ROS levels in bovine zygotes increase significantly at 7, 19, and 24 h after IVF, coinciding with high-energy cellular events, such as sperm penetration and nuclear decondensation, pronuclear formation, and the first mitotic division, respectively [50]. Similarly, bovine zygotes displayed peaks of oxygen consumption with a concomitant increase in ROS production at 7 and 24 h following IVF [51]. An increase in ROS production after sperm penetration has also been reported in murine [52] and Xenopus zygotes [53], suggesting that elevated ROS levels may be a conserved feature during zygote formation across species. Hence, given the inherent rise in ROS levels around syngamy, the possibility exists that this physiological increase in ROS production may have masked NEFA-induced changes in zygotic ROS expression.

A relevant technical feature to consider is the O_2_ level used during IVF, as it can influence ROS synthesis in preimplantation embryos, including zygotes. For instance, atmospheric O_2_ can induce more ROS formation than culture at 5% O_2_ in bovine zygotes [28]. As such, the use of atmospheric O_2_ during IVF in this study could have further concealed any possible effects of NEFA on ROS production in zygotes. However, the use of atmospheric O_2_ during bovine IVF is a standard practice as blastocyst formation is decreased when IVF is carried out under 5% O_2_ [54]. Conversely, in vitro culture of preimplantation cattle embryos in 5–6% O_2_ is a conventional approach due to its ability to promote blastocyst development [55,56]. This low O_2_ microenvironment and the relatively constant levels of ROS production during the cleavage stages (i.e., 2-to-8-cell stage) [57,58,59] suggest that subtle stress-induced increases in ROS may be more readily detected during early embryo development than during zygote formation. Indeed, previous research showed that oocyte maturation under high NEFA concentrations can increase ROS levels in resultant embryos with four or more cells [60]. Still, the impact of NEFA on ROS production in reproductive cells appears contradictory. For instance, Sutton-McDowall et al. [7] reported that bovine oocytes exposed to a mix of stearic, palmitic, and oleic acid during IVM displayed decreased ROS generation, whereas Marei et al. [61] found that oocytes matured under the same NEFA mix (including concentrations) showed an increase in ROS levels.

The contrasting results could be partially related to the analytical assays used to detect ROS levels. Sutton-McDowall et al. [7] employed peroxyfluor-1, a fluorescent probe specific for hydrogen peroxide [62]. In contrast, Marei et al. [61] used 2′,7′-dichlorodihydrofluorescein diacetate (DCFH-DA), which detects a broader range of ROS molecules, including hydrogen peroxide, hydroxyl radical and peroxyl radical [63]. The fluorescent ROS probe used in the present study was CellROX Green which detects hydroxyl radical and superoxide anion [64,65]. CellROX Green has been used in bovine zygotes, where an increase in ROS production was detected when zygotes were exposed to heat stress [28] or aflatoxins [66] after IVF. However, to the best of the authors’ knowledge, there are no studies analysing stressors exclusively during IVF with concomitant measurement of ROS production in resultant bovine zygotes.

The question remains as to how NEFA may interfere with the ability of matured oocytes to achieve fertilisation. Lipid accumulation in oocytes does not seem to be a likely cause as the lipid content did not increase following NEFA exposure during IVF [21]. Another aspect to consider is the effect of NEFA on the function of cumulus cells. For instance, since cumulus cells can select spermatozoa with a higher fertilisation potential in vitro [67,68,69] and they are susceptible for damage from high NEFA concentrations [7,70], it is reasonable to speculate that impaired cumulus cell activity may have contributed to the reduced fertilisation rate observed in the present experiment. However, studies investigating the capacity of cumulus cells to select spermatozoa under high NEFA concentrations are not available.

The reduced fertilisation rate may also be attributed to impaired spermatozoa quality observed in the present study. The lower proportion of spermatozoa with intact plasma membranes observed following exposure to high NEFA concentrations is consistent with findings from a previous study [21]. The plasma membrane of spermatozoa plays a crucial role in functional modifications required for fertilisation, including sperm motility, capacitation, and the acrosome reaction [71,72]. Indeed, previous studies showed that bovine spermatozoa with disrupted plasma membranes displayed low motility [73,74], supporting the findings of Desmet et al. [21], in which spermatozoa exposed to high NEFA concentrations exhibited increased plasma membrane damage and decreased motility. The low mitochondrial membrane potential detected in this study under high NEFA concentrations could also affect sperm motility, as studies have shown that bovine spermatozoa with low mitochondrial membrane potential had low sperm motility [74,75,76,77]. The same association has been reported in humans [78,79,80,81].

Our findings also show that the proportion of spermatozoa with an intact acrosome following exposure to high NEFA concentrations was increased, which contrasts with a previous study that reported no effect of NEFA exposure in the percentage of bovine spermatozoa with reacted acrosomes [21]. These conflicting results could be due to the omission of heparin in the IVF medium used in the previous study [21]. In cattle, heparin induces capacitation of spermatozoa rather than directly triggering the acrosome reaction [24], and capacitation is a prerequisite for the acrosome reaction [82,83,84]. Albeit speculative, it is possible that the omission of a capacitating agent precluded the detection of treatment-induced differences in the percentage of spermatozoa displaying acrosome exocytosis. In the absence of heparin, fewer than 10% of bovine spermatozoa would be expected to undergo spontaneous acrosome exocytosis [85]. However, the percentage of acrosome-reacted spermatozoa was not reported in the previous study examining the effects of high NEFA exposure on spermatozoa quality [21]. During in vitro capacitation (e.g., in the presence of heparin), a modest percentage of spermatozoa may undergo spontaneous acrosome reaction. In cattle, approximately 1–30% spermatozoa can display spontaneous acrosome reaction after 4 h of in vitro incubation in capacitating medium [38,86,87], which is generally consistent with the values observed in the control groups of the present study.

In vitro experiments in mice [88], cattle [89,90], and humans (including incubation of human sperm with transgenic mouse oocytes expressing human zona pellucida proteins) [91,92] have shown that the acrosome reaction is initiated when spermatozoa come into contact with the zona pellucida. Hence, when sperm undergo the acrosome reaction prematurely, before reaching the oocyte, their capacity to bind to the zona pellucida and fertilise the oocyte is impaired [93]. However, the role of the zona pellucida as the trigger of the acrosome reaction has been a matter of debate because although it is clear that the acrosome reaction is essential for fertilisation [94,95], in mice, it was found that the acrosome reaction can occur before the spermatozoa reach the zona pellucida, and these acrosome-reacted spermatozoa can achieve fertilisation [96]. Moreover, murine acrosome-reacted spermatozoa recovered from the perivitelline space are capable of penetrating the zona pellucida for a second time and producing live offspring [97], challenging the notion that mouse spermatozoa with a spontaneous acrosome reaction possess a decreased fertilising capacity [98]. Nevertheless, in human IVF studies, it has been shown that a high rate of spontaneous acrosome reaction is linked to a lower fertilisation rate [99,100,101,102]. Therefore, the higher proportion of acrosome-reacted sperm observed in the high-NEFA group may contribute to the reduced fertilisation rates observed in this group. From an in vivo perspective, the alterations in the acrosome, plasma membrane, and mitochondrial membrane potential of spermatozoa caused by high NEFA concentrations could impair pregnancy establishment. Indeed, artificial insemination with bovine semen containing a higher proportion of spermatozoa with intact plasma membrane [103,104,105], intact acrosome [103,105], and high mitochondrial membrane potential [103] has been associated with increased pregnancy rates.

The results of this study also indicate that a high NEFA microenvironment can increase DNA fragmentation of ejaculated spermatozoa. Notably, the data also revealed that the mitochondria in sperm exposed to elevated NEFA were more depolarised, as determined by ratiometric imaging of JC-1. Although we did not measure total mitochondrial activity, the indication of loss of mitochondrial polarity may explain the increased DNA fragmentation seen in sperm exposed to a high NEFA environment since the loss of mitochondrial polarity is a recognised source of ROS [106]. Elevated ROS production is a major source of oxidative stress, resulting in DNA damage [107]. This could contribute not only to failed fertilisation but also to the decreased blastocyst formation observed in the present study. Indeed, studies in cattle [108,109] and humans [78,110,111,112,113,114,115,116] have found that the likelihood of successful fertilisation during standard IVF decreases as the level of DNA damage increases in spermatozoa. However, spermatozoa with impaired DNA integrity can fertilise the oocyte [22], which partially explains the lack of association between DNA fragmentation in spermatozoa and fertilisation rates reported in human studies with conventional IVF [101,117,118,119,120,121]. Still, even though spermatozoa with DNA damage can achieve fertilisation, the resultant embryos have an impaired ability to develop to the blastocyst stage, as reported in IVF studies in both cattle [22,108] and humans [101,117,119]. These in vitro findings are supported by in vivo evidence, as a recent meta-analysis has shown a strong negative association between DNA fragmentation in spermatozoa and pregnancy rate following artificial insemination in farm animals, including cattle [122]. In human IVF, the impact of spermatozoal chromatin fragmentation on pregnancy rate following embryo transfer remains controversial, as some studies reported lower pregnancy rates with embryos derived from spermatozoa exhibiting elevated DNA fragmentation, whereas others found no significant association. A 2021 meta-analysis of 15 human studies reported that spermatozoa DNA damage negatively affects pregnancy rates, but this relationship lost significance after adjusting for publication bias [123]. Recent human IVF studies have failed to identify an influence of DNA damage in spermatozoa on pregnancy rates [115,117,118,121,124,125], but some have revealed a negative association with pregnancy loss [118,126]. The inconsistencies observed across studies are partly attributable to limited control over maternal factors, such as age, nutritional status, lifestyle (e.g., smoking, alcohol consumption, stress), ovarian reserve, hormonal environment, and uterine receptivity. This limitation is particularly relevant in human studies where the oocyte donor and the embryo recipient are typically the same individual, making it difficult to isolate paternal contributions. Variability in sperm parameters beyond DNA fragmentation may also influence outcomes but is often insufficiently controlled. In addition, technical factors, such as the type of DNA fragmentation assay, culture media composition, the duration of embryo culture (which determines the developmental stage at transfer), the use of rescue intracytoplasmic sperm injection (ICSI), and type of transfer (fresh vs. frozen), vary considerably between laboratories. Together, these maternal, paternal, and technical variables influence pregnancy outcomes and complicate efforts to clearly define the impact of spermatozoa DNA damage in human IVF.

The reduced blastocyst formation found in the High-NEFA group could have been further exacerbated by the delayed loss of H3K27me3 during the 2-cell to 4-cell transition. H3K27me3 is a repressive histone mark that it is present in the maternal pronucleus and absent in the paternal pronucleus at the time of fertilisation [127,128]. In bovine embryos, H3K27me3 levels progressively decline during the initial embryonic divisions, reaching minimal levels by the 8-to-16-cell stage, followed by the re-establishment of expression by the blastocyst stage [29,129,130,131,132]. A similar pattern is observed in human embryos, where H3K27me3 expression is nearly undetectable by the 8-cell stage but re-emerges during the transition from morula to blastocyst [133,134,135,136]. In mice, the evidence is less consistent. Some studies report that embryos retain H3K27me3 up to the 8-cell stage, with levels declining at the morula stage and reappearing at the blastocyst stage [132,136,137], while one study observed a pattern more similar to that seen in cattle and humans [138]. The decrease in H3K27me3 during early embryonic development in cattle is a cell cycle-independent event, involving lysine demethylase 6B (KDM6B), also known as Jumonji domain-containing protein 3 (JMJD3), which mediates the demethylation of H3K27me3 [29]. Chung et al. [139] demonstrated that gene knockdown and catalytic inhibition of KDM6B led to the production of 8-cell bovine embryos with high expression of H3K27me3, which resulted in a decrease in blastocyst formation. Hence, it is believed that in bovine embryos, the decrease in H3K27me3 is linked with a sound embryonic genome activation (EGA) that promotes development to the blastocyst stage and the establishment of a pluripotent embryonic epigenome [128,139].

Given that the erasure of H3K27me3 in cattle embryos results from active demethylation mediated by KDM6B rather than from dilution of methylation marks through cell division [29,139], delayed embryos at the 2-cell stage would be expected to display expression levels similar to those of 4-cell embryos following a normal developmental trajectory. Indeed, it has been reported that 2-cell and 4-cell bovine embryos examined at 40 hpf showed no differences in H3K27me3 abundance [39]. To the best of the authors’ knowledge, this is the first study to show that high NEFA concentrations during zygote formation can affect the expected decrease in H3K27me3 levels during the early cell divisions of bovine preimplantation embryos. However, since no difference in H3K27me3 levels was found between treatment groups in 4-cell embryos 48 hpf, the effect of high NEFA exposure on H3K27me3 demethylation during IVF appears to be subtle in the present in vitro model.

The decreased blastocyst production observed in this study contrasts with findings from a previous study, which reported no impact of NEFA exposure during IVF on blastocyst formation [21]. This discrepancy could be explained by differences in the embryo culture media used, as in the previous study, the culture medium was supplemented with insulin, transferrin, and selenium (ITS). ITS can enhance blastocyst production in cattle and is used in combination with BSA as a substitute for serum in embryo culture media [140]. Given that ITS can facilitate development to the blastocyst stage, it is possible that the detrimental effects of NEFA exposure during IVF on blastocyst formation were masked by ITS supplementation. Indeed, ITS supplementation can restore competence in oocytes matured in vitro under high NEFA concentrations, with resulting zygotes exposed to ITS reaching blastocyst formation rates similar to those of controls. However, embryo quality remained compromised, as indicated by increased apoptosis observed in the resultant blastocysts. These findings suggest that while ITS supports blastocyst formation, it does not seem to improve the quality of blastocysts derived from oocytes exposed to a high NEFA microenvironment during maturation [141]. The decreased cell numbers in both the ICM and TE in blastocysts derived from exposure to high NEFA concentrations during IVF in the present study also differ from the findings of Desmet et al. [21], who reported an increased TE cell number without affecting the ICM. These contrasting results could also be due to the use of ITS, as ITS supplementation has been shown to increase blastocyst cell numbers [140], including in embryos derived from NEFA-exposed oocytes [141]. Interestingly, the increase in TE without a change in ICM reported by Desmet et al. [21] did not correspond to an increase in the total cell number. Although speculative, this could be related to differences in methodology, as the previous study used fluorescence microscopy, which allows only two-dimensional visualisation of embryos [21], whereas the present study employed confocal microscopy, enabling three-dimensional visualisation and more detailed exploration of blastocysts, especially in dense regions such as the ICM.

The altered cell allocation in bovine blastocysts derived from the High-NEFA group could negatively impact the potential of embryos to undergo elongation and attach to the uterus. For instance, the blastocyst cell number has been shown to correlate with conceptus length on day 14 of pregnancy, with blastocysts with fewer cells producing shorter conceptuses [142]. Proper elongation of the conceptus trophectoderm is believed to be crucial for successful placentation and maintenance of pregnancy [143]. Along with the cell number, the allocation of ICM and TE cells is important for pregnancy success. In vivo studies have indicated that an ICM/TCN proportion between 20% and 40% falls within the normal range in bovine blastocysts [144,145,146]. Proportions above 40% have been reported in bovine somatic cell nuclear transfer (SCNT) embryos [145,147,148,149,150], and this impaired cell allocation has been associated with high rates of embryonic loss during early pregnancy (first trimester) after embryo transfer of SCNT embryos [145]. Hence, the presence of blastocysts in the High-NEFA group with very high ICM/TCN proportions could increase the risk of pregnancy loss.

Overall, the present in vitro model suggests that exposure to high NEFA concentrations during fertilisation can compromise fertilisation success, blastocyst formation and cell lineage allocation, which may negatively impact pregnancy outcomes. This detrimental effect appears to be mediated through both the oocyte and the sperm. Given that in a previous study, blastocyst formation was not affected with spermatozoa pre-exposed to high NEFA, even though plasma membrane integrity and motility were impaired, it was suggested that in a high NEFA microenvironment the fertilisation process may not be affected via the spermatozoa but rather through the oocyte [21]. However, in the study by Desmet et al. [21], the swim-up technique was used during NEFA exposure, which is essentially a spermatozoa selection method. Therefore, the possibility exists that damaged spermatozoa were removed before IVF, potentially masking detrimental effects associated with impaired spermatozoa quality. It is important to recognise that analysing the fertilisation potential of NEFA-exposed spermatozoa in a standard IVF setting is challenging, as the sperm suspension must be washed to remove NEFA before incubation with untreated oocytes, and the washing procedure could remove spermatozoa damaged by NEFA exposure. To address this issue, an ICSI model could offer a more accurate evaluation system. This approach would be particularly useful for examining the detrimental effects of high NEFA exposure on DNA fragmentation in spermatozoa. Future studies should also examine the individual contributions of specific NEFAs, perform transcriptomic analysis around the time of EGA, and assess ROS levels after the zygote stage to provide more mechanistic insights into the effects of NEFA at the time of fertilisation.

## 5. Conclusions

The results of this in vitro model suggest that when fertilisation takes place in a lipotoxic NEFA microenvironment, the ability of the spermatozoon to reach the oocyte is compromised due to decreased mitochondrial membrane potential and damage to the plasma membrane, both of which impair motility. The ability to attach to and penetrate the oocyte is also affected, as a result of detrimental effects on the plasma membrane and the acrosome. When affected spermatozoa manage to fertilise the oocyte, the resulting zygote would struggle to cleave and reach the blastocyst stage, likely due to DNA damage in the spermatozoa. The capacity of zygotes to proceed to the blastocyst stage could be further compromised by a putative delay in the activation of developmentally important genes at the time of embryonic genome activation, potentially resulting from a slower erasure of repressive epigenetic marks, such as H3K27me3. Furthermore, impaired cell allocation in the resulting blastocyst could further jeopardise pregnancy success. Hence, taking into consideration, the present findings and previous research on oocyte maturation and early embryo development, it can be inferred that under natural conditions, high NEFA concentrations may affect oocyte competence (i.e., before fertilisation), the fertilisation process, and preimplantation embryo quality (i.e., after fertilisation). Consequently, the cumulative detrimental effects of high NEFA exposure on these key developmental milestones are likely to contribute significantly to the infertility observed in cattle experiencing NEB and possibly in obese individuals as well.

## Data Availability

The original contributions presented in this study are included in the article. Further inquiries can be directed to the corresponding author.
